# Investigating the effect of SGLT2 inhibitors on cardiovascular related health status in HFmrEF and HFpEF: systematic review and meta analysis

**DOI:** 10.3389/fcvm.2025.1556606

**Published:** 2025-07-04

**Authors:** Tabitha Kusi-Yeboah, Isaac Gianfrancesco, Muzammil Arif Din Abdul Jabbar, Phoebe Collins, Dalton James Bally, Juliet Thornton, Kieran Williams, Ayoola Ishola, Lucy Hong, Ping Jing Toong, Milindu Wickramarachchi

**Affiliations:** ^1^ Clinical School, University of Cambridge, Cambridge, United Kingdom; ^2^ Clinical School, University College London, London, United Kingdom

**Keywords:** SGLT2 inhibitors (SGLT2i), heart failure, HFmrEF, HFPEF, quality of life, cardiovascular health, systematic review, meta-analysis

## Abstract

**Background and aims:**

Sodium-glucose co-transporter 2 inhibitors (SGLT2i) have emerged as an integral component of heart failure management, with evidence supporting their benefits across a broad spectrum of ejection fractions. However, their impact on quality of life (QoL) in patients with heart failure with mildly reduced (HFmrEF) and preserved ejection fraction (HFpEF) remains underexplored. This systematic review and meta-analysis aim to evaluate the effects of SGLT2i on QoL compared to standard therapy in these patient populations.

**Methods:**

A systematic search of PubMed, Cochrane, and EMBASE databases was conducted for randomized controlled trials (RCTs) published in English that assessed the clinical outcomes of SGLT2i in HFpEF and HFmrEF up to January 23, 2024. Two independent reviewers evaluated the risk of bias for eligible studies. A random-effects model was used for meta-analysis. The primary outcomes of interest were changes in the Kansas City Cardiomyopathy Questionnaire (KCCQ) score and 6 Minute Walk Test Distance (6MWTD).

**Findings:**

A total of 7 RCTs comparing KCCQ score in HFpEF and HFmrEF in participants receiving SGLT2i vs. placebo, and 3 RCTs comparing 6MWTD in HFpEF and HFmrEF in participants receiving SGLT2i vs. placebo were included in the systematic review. Overall SGLT2i was associated with an increase in KCCQ-TSS score (MD = 2.28, 95% CI 1.94–2.63, *I*^2^ = 0%) and 6MWTD (MD = 13.52, 95% CI 1.70–25.34, *I*^2^ = 62%).

**Interpretation:**

These findings suggest that SGLT2i not only confer cardiovascular benefits but also enhance patient-reported health status, reinforcing their role as a valuable adjunct to standard heart failure therapy in HFmrEF and HFpEF.

## Introduction

Chronic heart failure (CHF) is characterized by structural or functional impairment in ventricular filling or ejection of blood, as defined by the American College of Cardiology (1). Heart failure (HF) remains a significant global health challenge, affecting an estimated 64.3 million individuals worldwide, with prevalence projected to rise due to the aging population (2). Based on left ventricular ejection fraction (LVEF), CHF is classified into four subtypes: heart failure with reduced ejection fraction (HFrEF) with LVEF ≤40%; heart failure with mildly reduced ejection fraction (HFmrEF) with LVEF 41%–49%; heart failure with preserved ejection fraction (HFpEF) with LVEF ≥50%; and heart failure with improved ejection fraction (HFimpEF), characterized by a baseline LVEF of ≤40% with subsequent improvement to >40% (3).

The primary goals of heart failure management are to reduce cardiovascular mortality and improve quality of life by alleviating symptoms and minimizing functional limitations. Standard pharmacological management includes a combination of angiotensin-converting enzyme inhibitors (ACEIs) or angiotensin receptor blockers (ARBs), beta-blockers, and mineralocorticoid receptor antagonists (MRAs). Recent evidence from multiple clinical trials has highlighted the significant benefits of sodium-glucose co-transporter 2 inhibitors (SGLT2i), originally developed as anti-diabetic agents, in the management of HF (4). These studies have demonstrated that SGLT2i therapy reduces cardiovascular-related hospitalizations, mortality, and symptom burden in patients with HFrEF (5–7). However, fewer studies have evaluated the impact of SGLT2i in patients with HFmrEF and HFpEF. Nevertheless, emerging data suggest that SGLT2i confer similar benefits in these patient groups (8).

Numerous SGLT2 inhibitors have been evaluated in extensive cardiovascular outcome trials, each exhibiting distinct pharmacological profiles and demonstrating consistent clinical benefits across varied populations.

Empagliflozin was the inaugural agent in this class to exhibit cardiovascular benefits in the EMPA-REG OUTCOME trial (9), demonstrating a significant reduction in cardiovascular mortality and hospitalization among patients with type 2 diabetes and established atherosclerotic cardiovascular disease. Subsequently, dapagliflozin showed notable advantages in heart failure-related endpoints in the DAPA-HF and DELIVER trials (10). Although primarily assessed in the context of type 2 diabetes in the VERTIS-CV trial, ertugliflozin has similarly demonstrated a favorable cardiovascular safety profile (11, 12). Based on this body of evidence, the FDA approved empagliflozin for heart failure in 2021, followed by dapagliflozin in 2022. These regulatory milestones highlight the evolving role of SGLT2 inhibitors beyond glycemic control, establishing them as foundational therapies in cardiovascular medicine. While all SGLT2 inhibitors share a common mechanism of action, namely the inhibition of glucose reabsorption in the proximal renal tubule, their effects on volume status, neurohormonal modulation, and inflammation vary slightly based on individual pharmacodynamics (13, 14). This has led to increased interest in characterizing their impact not only on hard cardiovascular outcomes (e.g., mortality and hospitalization) but also on patient-centred endpoints such as health-related quality of life (QoL) (15).

A limited number of studies have examined the effects of SGLT2i on patient-reported quality of life (QoL) outcomes in HFmrEF and HFpEF. Current clinical guidelines from the European Society of Cardiology (ESC) and the American Heart Association (AHA), American College of Cardiology (ACC), and Heart Failure Society of America (HFSA) have included SGLT2i as a class 1a recommendation for HFrEF and a class 2a recommendation for HFmrEF/HFpEF to reduce cardiovascular death and hospitalization (16, 17). Additionally, the ESC guidelines endorse SGLT2i for improving symptom burden and QoL in patients with HFrEF (18). However, the extent to which these benefits extend to HFmrEF and HFpEF remains unclear or unaddressed in updated guidelines (17).

As SGLT2 inhibitors (SGLT2i) are increasingly utilized for broader indications, encompassing the entire spectrum of ejection fraction, it is imperative to further elucidate their role in heart failure with mid-range ejection fraction (HFmrEF) and heart failure with preserved ejection fraction (HFpEF). While clinical trials such as EMPEROR-Preserved and DELIVER have begun to address this knowledge gap by demonstrating improvements in both cardiovascular outcomes and quality of life metrics, the real-world applicability and drug-specific differences remain subjects of ongoing investigation (19, 20).

This systematic review aims to provide a comprehensive analysis of studies investigating the role of SGLT2 inhibitors in improving QoL in patients with HFmrEF and HFpEF, regardless of diabetic status. The review focuses on studies reporting QoL outcomes, highlighting the evidence supporting SGLT2i as a potential adjunct to standard heart failure therapy in these populations.

## Methods

### Eligibility criteria

Our study aimed to find, assess, and synthesize all randomized controlled trials (RCTs) containing symptomatic chronic heart failure patients with preserved and mildly reduced ejection fraction. Articles were excluded in the case of: (1) Studies investigating acute heart failure; (2) Studies investigating chronic heart failure with ejection fraction <40%; (3) Outcomes were reported in special populations, including NYHA class, gender, age, frailty and ethnicity; (4) Studies investigating SGLT2 inhibitor use in populations other than HFmrEF and/or HFpEF (5) Duplicates; (6) Outcome of interest not reported. Interventions included SGLT2 inhibitors. Comparators were patients with HFmrEF and/or HFpEF receiving standard treatment for chronic heart failure (ACE/ARB + Beta Blockers +/- MRA).

### Search strategy

A comprehensive search of PubMed, Embase and Cochrane databases was performed to include articles from the databases' date of inception to January 23rd, 2024. The following components were included in the search string: Medical Subject Headings (MeSH terms) in combination with the terms “Chronic Heart Failure” and “SGLT-2 inhibitors”. A detailed search strategy is provided in Supplementary Material.

### Study selection and data extraction

The title and abstracts were reviewed in the first screening phase by TKY. Duplicates were removed, and successful studies were then subject to full-text review. Two investigators (DB/PJT, KW/MJ, PC/MJ, IG/AI, MW/LH, JT/IG) independently reviewed the eligibility of the retrieved full-text studies. If there were any discrepancies, reasons for exclusion were noted and discussed with TKY as the mediator until a decision was reached.

### Outcomes of interest

Outcomes of interest included: (1) Change in health status assessed via the Kansas City Cardiomyopathy Questionnaire (KCCQ) (21), a heart failure specific tool quantifying symptom frequency, symptom stability, symptom burden, physical limitations, quality of life, social limitations and self-efficacy within a 2-week recall period based on 23 individual factors, including KCCQ-Total symptom score (KCCQ-TSS), KCCQ-Clinical symptom score (KCCQ-CSS), and KCCQ-Overall symptom score (KCCQ-OSS); (2) Changes in 6 Min walk test distance from baseline. Studies investigating hospitalization rates as the sole outcome in HFmr/pEF after SGLT2i administration were excluded. Studies that did not provide full text or relevant data in their abstract, and studies not written in English were also excluded.

### Assessment of quality and risk of bias

Two reviewers (DB/PJT, KW/MJ, PC/MJ, IG/AI, MW/LH, JT/IG) independently reviewed each included study for risk of bias using the Revised Cochrane risk-of-bias tool for Randomised trials (RoB 2), recommended for quality assessment of RCTs (22). Discrepancies were discussed and resolved between reviewers. Using 5 domains the overall risk of bias for each included study was assessed. Studies were judged as low risk if there was a low risk of bias for all domains, as raising some concerns if at least one domain was flagged for this result but not as high risk for any domain, or judged to be high risk if high risk of bias was noted in at least one domain or the study was judged to have some concerns in multiple domains in a way that substantially lowered confidence in a result.

### Data synthesis and analysis

The mean difference with 95% confidence intervals was calculated for the desired outcomes, KCCQ-TSS, KCCQ-OSS, KCCQ-CSS, and 6MWTD. The quantile estimation method by McGrath et al. (23) was used to convert desired outcomes reported as median difference to mean difference. Data was combined in a systematic review, forest plot and meta-analysis. The meta-analysis was carried out using the random effects model. The analysis included the study of overall effect size and the existence of heterogeneity. The *Q* and *I*^2^ test were used to analyse heterogeneity, with *I*^2^ > 50% indicating notable heterogeneity. Subgroup and sensitivity analyses were performed when necessary to investigate sources of heterogeneity. Statistically significant results were identified with *p* < 0.05 and CIs excluding a null effect. The risk of publication bias was examined by visual inspection of funnel plots. Meta analysis was performed using Review Manager Software (version 5.4, The Cochrane Collaboration, Software Update, Oxford, UK).

## Results

**Figure 1:**
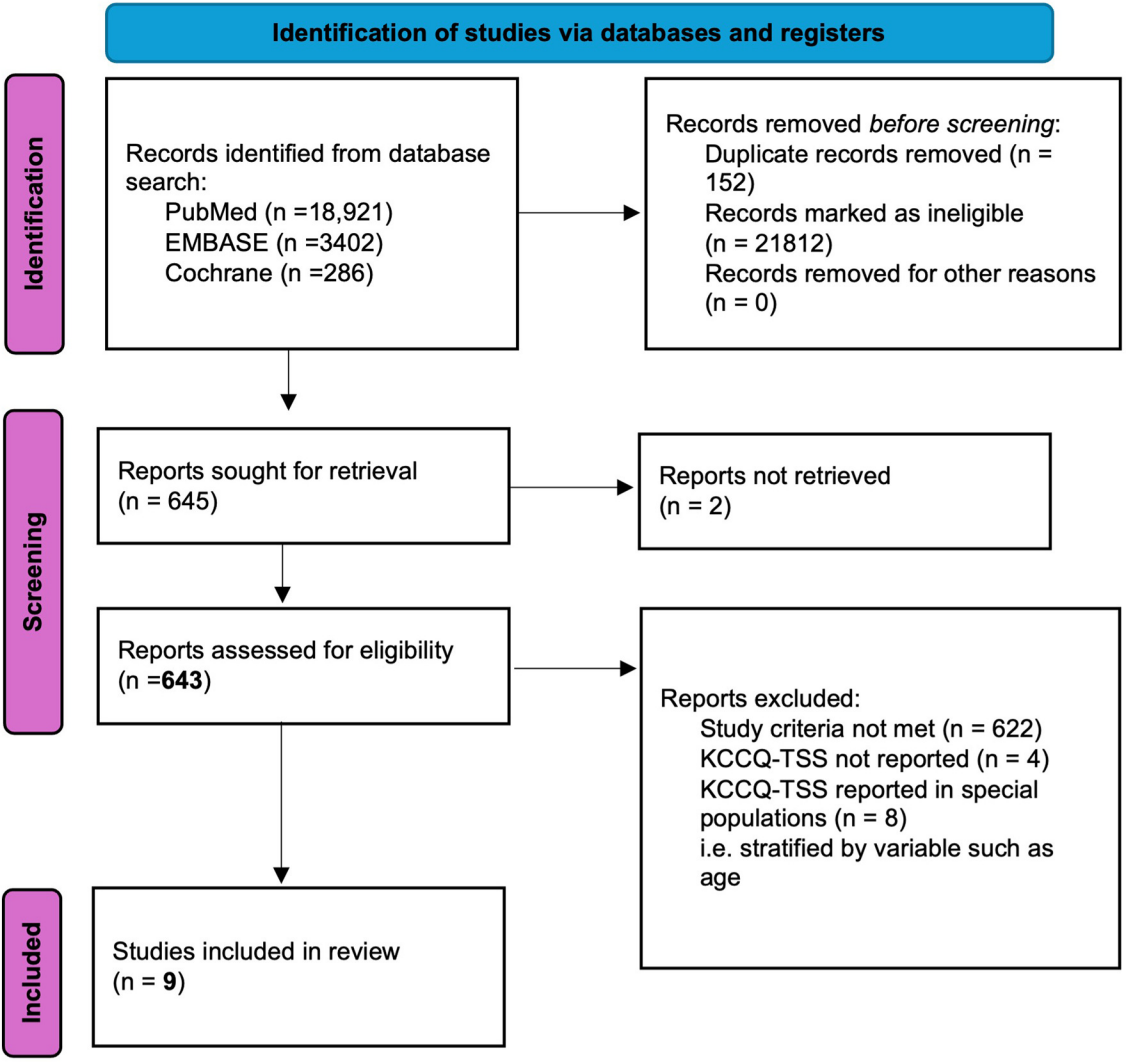
PRISMA flow diagram.

The database search revealed 22,609 articles. After removing duplicates, non-relevant and unretrievable articles, 643 studies remained for full text evaluation Figure 1. Of these, 634 articles were removed in the case of: (1) Studies investigating acute heart failure; (2) Studies investigating chronic heart failure with ejection fraction <40%; (3) Outcomes were reported in special populations, including NYHA class, gender, age, frailty and ethnicity; (4) Studies investigating STGL2i use in populations other than HFmrEF and/or HFpEF (5) Duplicates; (6) Outcomes of interest not reported. There remained 9 randomised controlled trials (RCTs) eligible for inclusion in quantitative analysis as seen in Figure 1 (24–32). Of which 7 were RCTs comparing the effect of SGLT2i vs. placebo on Kansas City Cardiomyopathy Questionnaire (KCCQ), and 3 were RCTs comparing the effect of SGLT2i vs. placebo on health status as assessed using 6 Min Walk Test Distance (6MWTD).

Among the reported randomised control trials (RCTs), seven investigate the effect of SGLT2i vs. placebo on KCCQ-TSS score. All seven RCTs report an increase in KCCQ-TSS in comparison to standard heart failure therapy. Spertus et al. (24) report the highest recorded mean change in KCCQ-TSS score from baseline relative to placebo.

Nassif et al. (25), Abraham et al. (26) and Lewis et al. (29) investigate the effect of SGLT2i vs. placebo on 6MWTD. Nassif et al. (25) and Lewis et al. (29) report statistically significant increases in 6MWTD from baseline in treatment groups in comparison to placebo groups at 12 weeks [20.1M (95% CI 5.6–34.7, *P* = 0.007); 20.1 (5.55, 34.69, 95% CI)].

### Study quality and risk of bias

**Figure 2:**
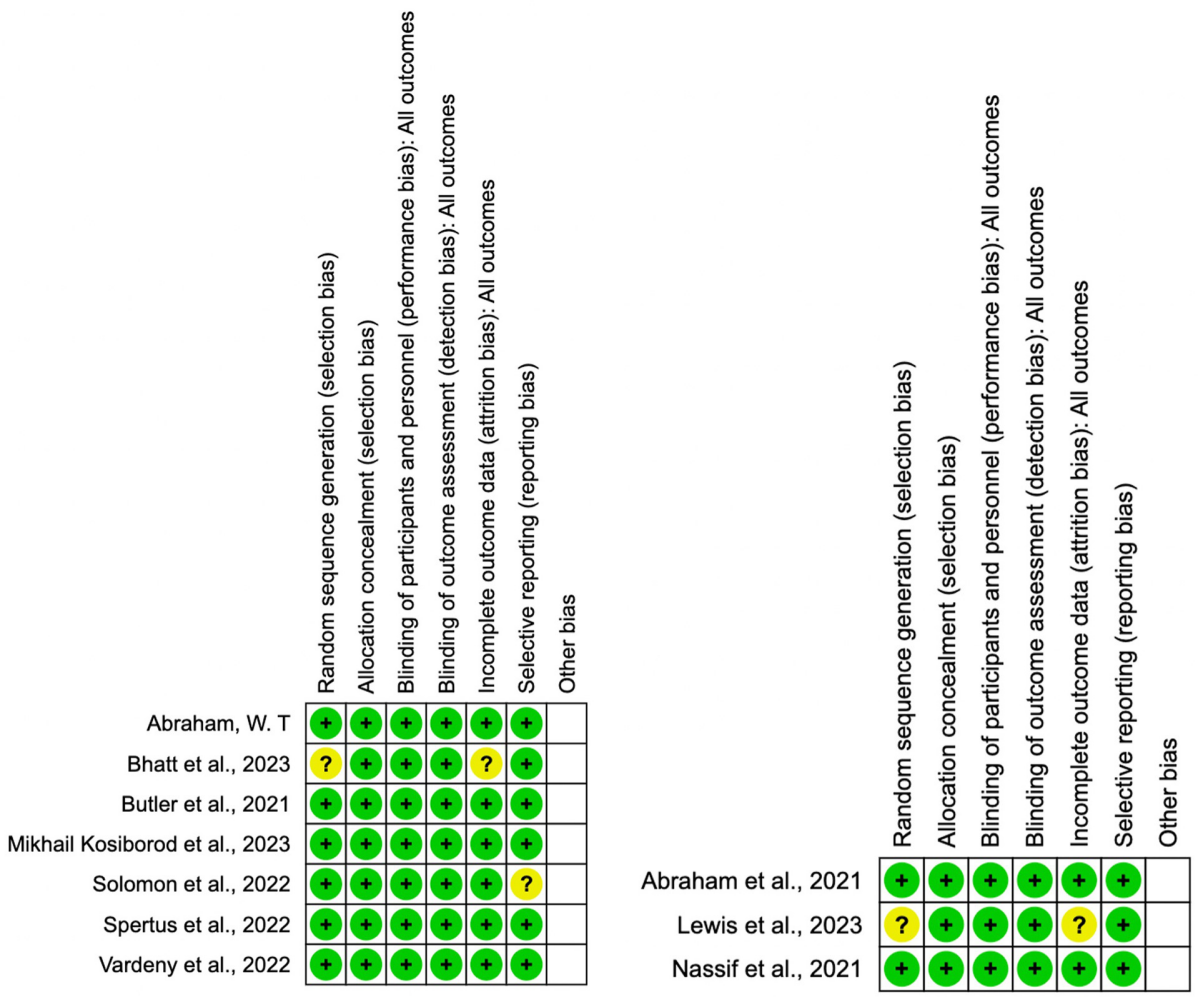
Risk of bias assessment for included RCTs using ROB2 tool.

Figure 2 displays results of the risk of bias analysis for included studies conducted using the Revised Cochrane risk-of-bias tool for Randomised trials (RoB 2). Six studies had an overall low risk of bias, and three studies had an overall risk of bias warranting “some concerns”.

## Clinical outcomes

### KCCQ-TSS

**Figure 3:**
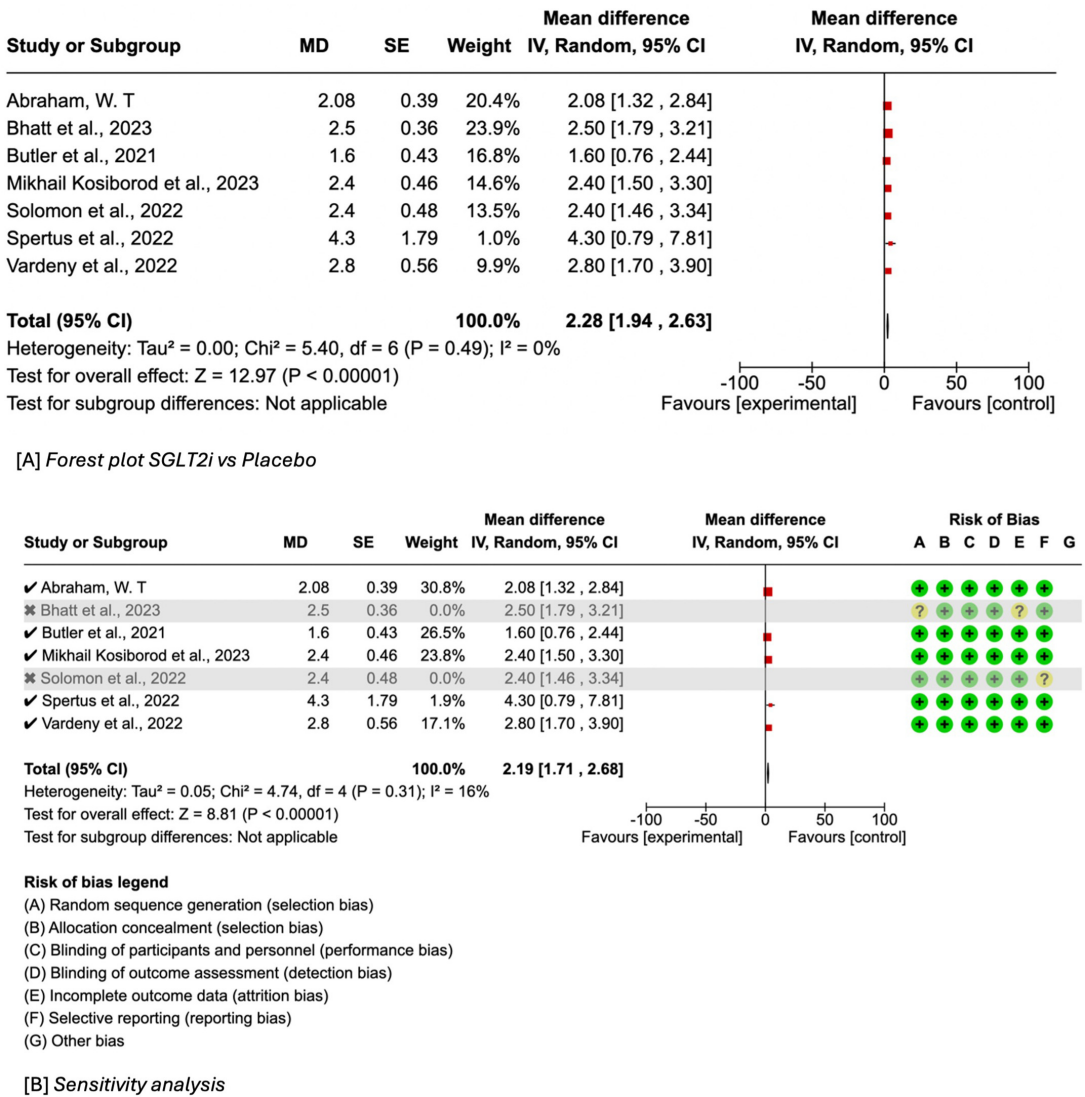
**(A)** Forest plot of mean difference in KCCQ-TSS score of SGLT2i versus placebo in HFmr/pEF. **(B)** Summary of sensitivity analysis of included studies.

Seven of nine included studies reported on KCCQ-TSS with SGLT2i (canagliflozin, empagliflozin or dapagliflozin) use. Hence in the meta-analysis, 12,511 received an SGLT2i and 12,598 patients received standard heart failure therapy. The use of SGLT2i was associated with a statistically significant increase in KCCQ-TSS score (MD = 2.28, 95% CI 1.94–2.63, *I*^2^ = 0%) as seen in Figure 3A. Upon sensitivity analysis by excluding studies with an overall risk of bias warranting “some concern” [Bhatt et al. (27) and Solomon et al. (31)] the results remained significant and continued to trend towards a benefit with SGLT2 inhibitors (MD = 2.19, 95% CI 1.71–2.68, *I*^2^ = 16%) (Figure 3B).

### KCCQ-OSS and KCCQ-CSS

One study, by Nassif et al. (25), reported a 4.5 point (95% CI 1.1–7.8 *P* = 0.009) increase in KCCQ-OSS from baseline relative to placebo with empagliflozin use. Nassif et al. (25) also report a 5.8 point (95% CI 2.3–9.2, *p* = 0.001) increase in KCCQ-CSS from baseline relative to placebo with empagliflozin use. These results were excluded from meta-analysis as an insufficient number of studies included in this systematic review report KCCQ-OSS and KCCQ-CSS scores.

## 6 Minute walk test distance

**Figure 4:**
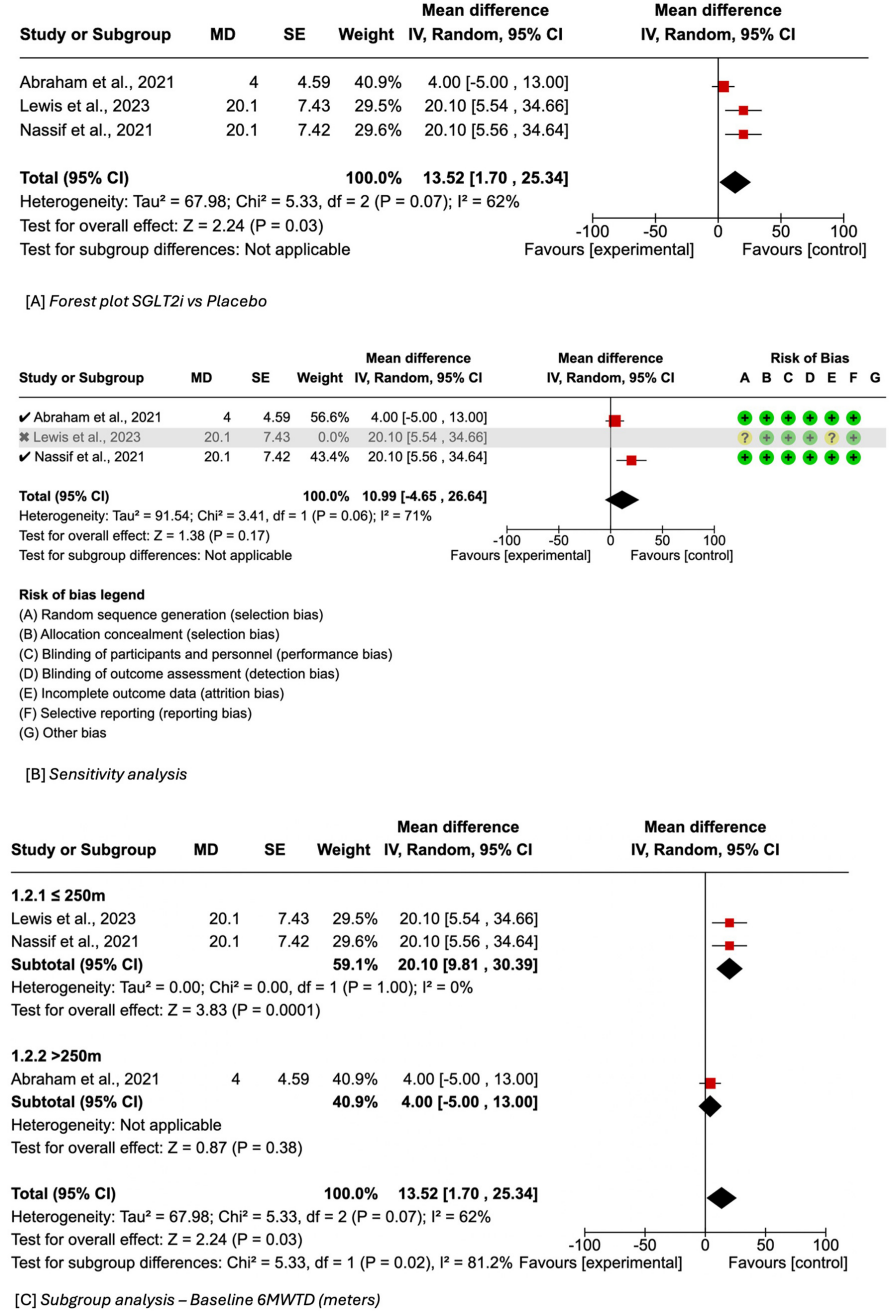
**(A)** Forest plot of mean difference in 6MWTD of SGLT2i versus placebo in HFmr/pEF. **(B)** Summary of sensitivity analysis of included studies. **(C)** Subgroup analysis according to baseline 6MWTD in meters.

Three out of nine studies reported 6 min walk test distance (6MWTD) with empagliflozin or dapagliflozin use. The meta-analysis showed that therapy with SGLT2i was associated with a statistically significant increase in 6MWTD in comparison to standard therapy (MD = 13.52, 95% CI 1.70–25.34, *I*^2^ = 62%) as shown in Figure 4A. Upon sensitivity analysis by excluding studies with an overall risk of bias warranting “some concern” [Lewis et al. (29)], the results became non-significant but continued to trend towards an increase in 6MWTD with SGLT2i use (MD = 10.99, 95% CI −4.65–26.64, *P* = 0.17, *I*^2^ = 71%) (Figure 4B). The random effects model analysis revealed a statistically significant difference in the effects on subgroup analysis between ≤250 m and >250 m (*P*-value for sub-group differences = 0.02) as shown in Figure 4C.

### Publication bias

**Figure 5:**
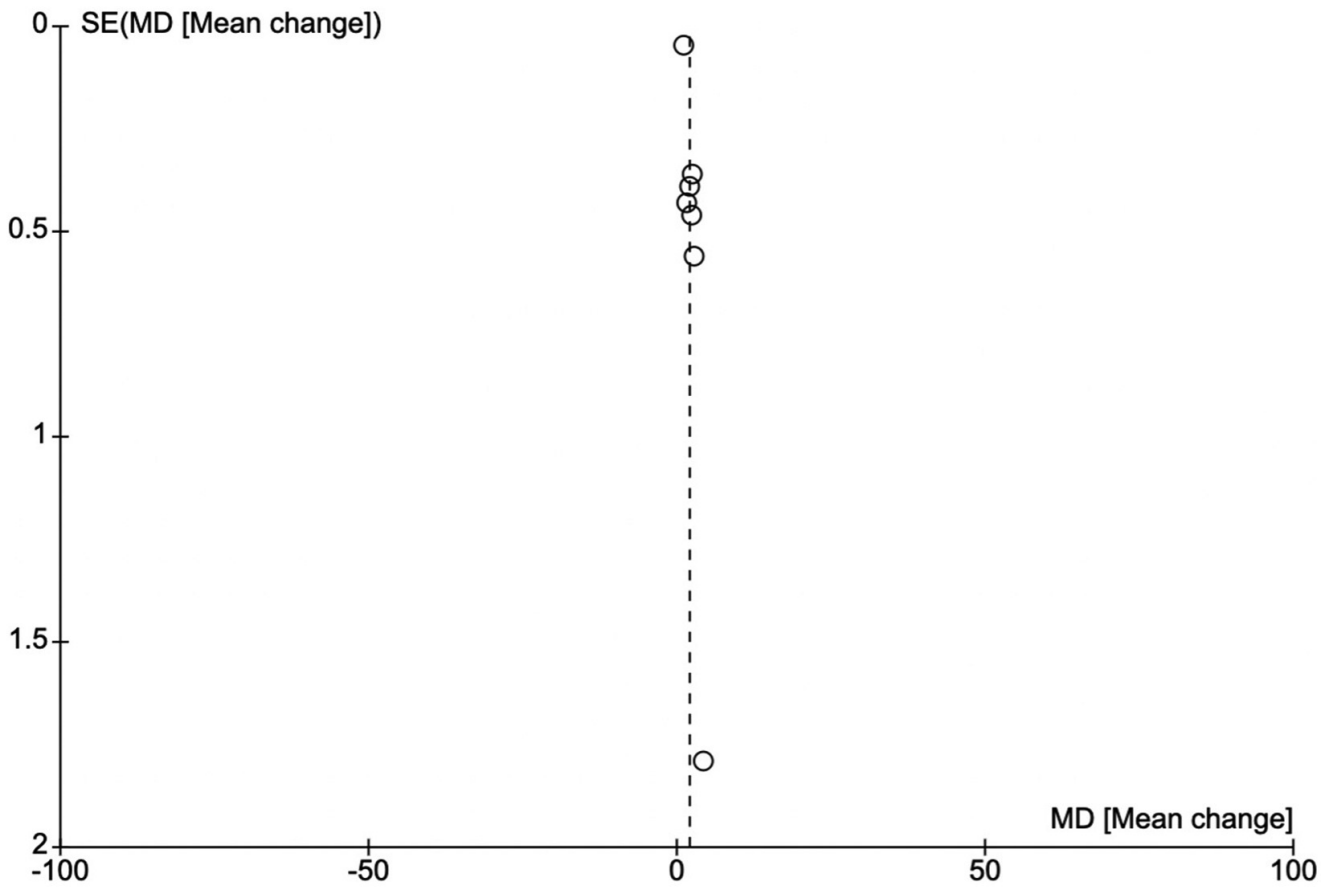
Funnel plot of KCCQ-TSS vs. standard therapy in HFmrEF or HFpEF.

**Figure 6:**
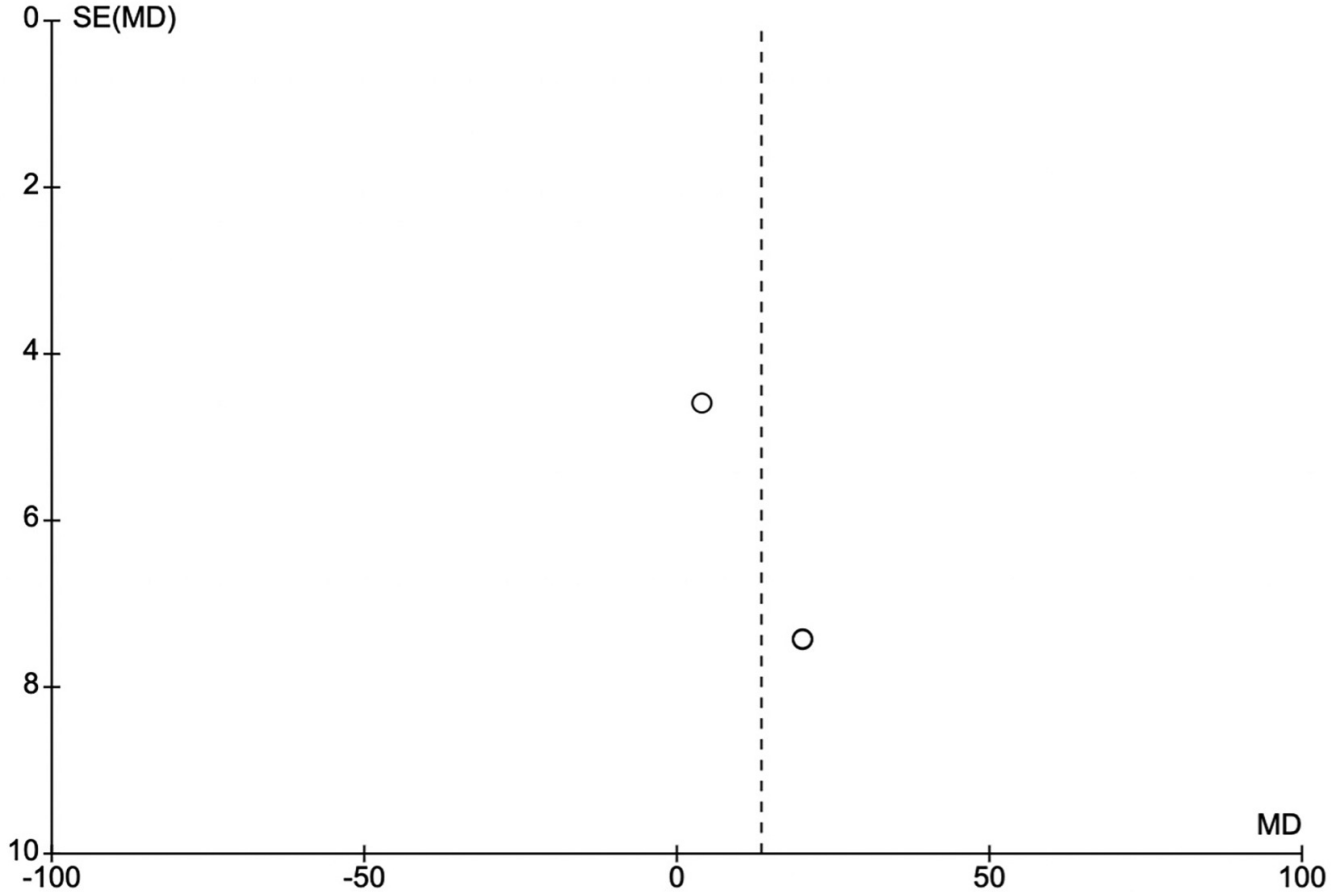
Funnel plot of 6MWTD vs. standard therapy in HFmrEF or HFpEF.

Funnel plots show symmetry around the line of no effect as shown in Figures 5, 6, indicating a low risk of publication bias in the meta-analysis.

## Discussion

Heart failure management has increasingly emphasized not only the reduction of mortality and hospitalizations but also the improvement of patient-reported outcomes, particularly in subtypes such as heart failure with mildly reduced ejection fraction (HFmrEF) and preserved ejection fraction (HFpEF). These populations, traditionally underserved in terms of evidence-based therapies, experience a significant symptom burden that can adversely affect functional capacity and quality of life (QoL) (33, 34). While the benefits of sodium-glucose co-transporter 2 inhibitors (SGLT2i) in heart failure with reduced ejection fraction (HFrEF) are well established, the therapeutic value of these agents in HFmrEF and HFpEF has only recently gained clarity (20).

This systematic review and meta-analysis aimed to address a critical knowledge gap by evaluating the effects of SGLT2i on QoL outcomes in HFmrEF and HFpEF. Our findings, which included over 25,000 participants across seven randomized controlled trials (RCTs), indicate that SGLT2i therapy is associated with a statistically significant improvement in Kansas City Cardiomyopathy Questionnaire Total Symptom Score (KCCQ-TSS), with a mean difference of 2.28 points (95% CI: 1.94–2.63, *I*^2^ = 0%). In addition, analysis of three RCTs assessing the 6 min walk test distance (6MWTD) demonstrated a modest but significant improvement (mean difference of 13.52 meters; 95% CI: 1.70–25.34, *I*^2^ = 62%). Subgroup analyses suggest that patients with lower baseline functional capacity may derive more pronounced benefit in 6MWTD.

While the primary emphasis of this review was on quality of life (QoL), numerous studies included in the analysis, notably EMPEROR-Preserved and DELIVER, also documented enhancements in cardiovascular outcomes, such as reductions in hospitalizations due to heart failure. These findings underscore the dual function of SGLT2 inhibitors (SGLT2i) in both altering disease progression and improving patient experience. Importantly, these advantages appear consistent across various agents, with empagliflozin and dapagliflozin providing substantial evidence for both prognostic and symptomatic improvement (35–37). Although ertugliflozin has been less extensively studied in heart failure with mid-range ejection fraction (HFmrEF) and heart failure with preserved ejection fraction (HFpEF), it has demonstrated cardiovascular safety in related populations (38). The uniformity of these results bolsters the argument for a class-wide benefit.

Despite the increasing clinical endorsement of SGLT2 inhibitors (SGLT2i), the exact mechanisms through which these agents enhance symptoms and functional outcomes remain a subject of ongoing research. Several interconnected hypotheses have been proposed. Firstly, SGLT2i exhibit a mild yet sustained diuretic and natriuretic effect, which reduces intravascular volume and left ventricular filling pressures without activating the renin–angiotensin–aldosterone system (39, 40). This may contribute to the alleviation of congestion-related symptoms such as dyspnea and peripheral edema, particularly in heart failure with preserved ejection fraction (HFpEF), where diastolic stiffness is prevalent.

Second, anti-inflammatory effects have been documented in numerous SGLT2i trials, with reductions observed in biomarkers such as IL-6 and TNF-α. These agents may modulate the systemic inflammatory state associated with HFpEF pathophysiology, thereby enhancing endothelial function and diminishing myocardial fibrosis (20, 41, 42). Third, SGLT2i appear to exert beneficial effects on autonomic regulation. Emerging evidence indicates that these drugs reduce sympathetic nervous system activity and enhance parasympathetic tone, as evidenced by increased heart rate variability and improved baroreflex sensitivity—factors that may correlate with enhanced exercise tolerance and subjective well-being (13, 43).

Furthermore, there is increasing interest in the role of SGLT2 inhibitors (SGLT2i) in enhancing myocardial energetics. By facilitating a shift in substrate utilization towards ketone bodies, which are more oxygen-efficient compared to glucose or fatty acids, SGLT2i may improve cardiac energy efficiency. This potential benefit is particularly relevant in heart failure with preserved ejection fraction (HFpEF), where a mismatch between myocardial oxygen delivery and demand is a contributing factor. Additionally, improvements in vascular compliance and arterial stiffness have been documented, which may further support peripheral oxygen delivery and exercise capacity (20, 39, 44).

While these multifactorial mechanisms offer a plausible explanation for the observed benefits, it is essential to acknowledge certain limitations within the current evidence base. Most notably, although the Kansas City Cardiomyopathy Questionnaire Total Symptom Score (KCCQ-TSS) was consistently reported, other significant domains of the KCCQ, such as the Clinical Summary Score (KCCQ-CSS) and Overall Summary Score (KCCQ-OSS), were not universally available. This limitation constrains our ability to assess more granular health status outcomes. Furthermore, although the mean change in KCCQ-TSS was statistically significant, it remains below the 5-point threshold commonly regarded as the minimal clinically important difference. Nonetheless, in the context of a chronic and symptomatic disease, even modest improvements may have meaningful implications for patients.

A further limitation concerns the diversity of study populations. The majority of participants were of Caucasian descent, with limited representation of racial and ethnic minorities. Given the established disparities in heart failure outcomes across different demographic groups, future trials should aim for more inclusive recruitment to enhance the generalizability of findings. Additionally, the heterogeneity in study durations, which varied from 12 weeks to 8 months, may have influenced the extent of QoL improvement observed, although sensitivity analyses did not indicate duration-dependent effects.

Despite these limitations, our findings align with the broader body of literature indicating that SGLT2i exert a multifaceted and clinically significant impact on patients with HFmrEF and HFpEF. These effects, in conjunction with previously established benefits in reducing cardiovascular morbidity and mortality, suggest that SGLT2i should be considered not only as a disease-modifying therapy but also as an agent capable of enhancing patients' lived experience of heart failure.

In conclusion, this meta-analysis presents compelling evidence that SGLT2 inhibitors enhance quality of life and functional status in patients with HFmrEF and HFpEF. These findings highlight the significance of incorporating patient-centered outcomes in clinical trial design and advocate for the continued integration of SGLT2i into guideline-directed medical therapy for heart failure. Future research should focus on elucidating the optimal choice of agent, treatment duration, and mechanisms of response, particularly in underrepresented populations and those with multimorbidity.

## Data Availability

The original contributions presented in the study are included in the article/Supplementary Material, further inquiries can be directed to the corresponding author.

## Supplementary material

Supplementary Table 1 Baseline characteristics of included RCTs.
