# Supplementary material for: Investigating the effect of SGLT2 inhibitors on cardiovascular related health status in HFmrEF and HFpEF: systematic review and meta analysis
Source: Front Cardiovasc Med. 2025 Jul 4;12:1556606. doi: 10.3389/fcvm.2025.1556606 (PMC12271201; doi:10.3389/fcvm.2025.1556606)
Supplement: Supplementary Table 1 — Baseline characteristics of included RCTs. [file Table1.docx]

| Database | Search Strategy | Hits |
| --- | --- | --- |
| PubMed | ((("sodium glucose transporter 2 inhibitors"[Pharmacological Action] OR "sodium glucose transporter 2 inhibitors"[MeSH Terms] OR "sodium glucose transporter 2 inhibitors"[All Fields] OR "sodium glucose transporter 2 inhibitors"[All Fields] OR ("sodium glucose transporter 2 inhibitors"[Pharmacological Action] OR "sodium glucose transporter 2 inhibitors"[MeSH Terms] OR "sodium glucose transporter 2 inhibitors"[All Fields] OR "sodium glucose transporter 2 inhibitors"[All Fields]) OR ("sodium glucose transporter 2 inhibitors"[Pharmacological Action] OR "sodium glucose transporter 2 inhibitors"[MeSH Terms] OR "sodium glucose transporter 2 inhibitors"[All Fields] OR "sglt 2 inhibitors"[All Fields]) OR ("sodium glucose transporter 2 inhibitors"[Pharmacological Action] OR "sodium glucose transporter 2 inhibitors"[MeSH Terms] OR "sodium glucose transporter 2 inhibitors"[All Fields] OR "sglt 2 inhibitors"[All Fields]) OR ("sodium glucose transporter 2 inhibitors"[Pharmacological Action] OR "sodium glucose transporter 2 inhibitors"[MeSH Terms] OR "sodium glucose transporter 2 inhibitors"[All Fields] OR ("sglt2"[All Fields] AND "inhibitors"[All Fields]) OR "sglt2 inhibitors"[All Fields]) OR ("sodium glucose transporter 2 inhibitors"[Pharmacological Action] OR "sodium glucose transporter 2 inhibitors"[MeSH Terms] OR "sodium glucose transporter 2 inhibitors"[All Fields] OR "sodium glucose transporter 2 inhibitor"[All Fields]) OR ("sodium glucose transporter 2 inhibitors"[Pharmacological Action] OR "sodium glucose transporter 2 inhibitors"[MeSH Terms] OR "sodium glucose transporter 2 inhibitors"[All Fields] OR "sodium glucose transporter 2 inhibitor"[All Fields]) OR ("sodium glucose transporter 2 inhibitors"[Pharmacological Action] OR "sodium glucose transporter 2 inhibitors"[MeSH Terms] OR "sodium glucose transporter 2 inhibitors"[All Fields] OR ("sglt2"[All Fields] AND "inhibitor"[All Fields]) OR "sglt2 inhibitor"[All Fields]) OR ("sodium glucose transporter 2 inhibitors"[Pharmacological Action] OR "sodium glucose transporter 2 inhibitors"[MeSH Terms] OR "sodium glucose transporter 2 inhibitors"[All Fields] OR ("inhibitor"[All Fields] AND "sglt2"[All Fields]) OR "inhibitor sglt2"[All Fields]) OR ("sodium glucose transporter 2 inhibitors"[Pharmacological Action] OR "sodium glucose transporter 2 inhibitors"[MeSH Terms] OR "sodium glucose transporter 2 inhibitors"[All Fields] OR "gliflozin"[All Fields] OR "gliflozins"[All Fields]) OR ("sodium glucose transporter 2 inhibitors"[Pharmacological Action] OR "sodium glucose transporter 2 inhibitors"[MeSH Terms] OR "sodium glucose transporter 2 inhibitors"[All Fields] OR "sglt 2 inhibitor"[All Fields]) OR ("sodium glucose transporter 2 inhibitors"[Pharmacological Action] OR "sodium glucose transporter 2 inhibitors"[MeSH Terms] OR "sodium glucose transporter 2 inhibitors"[All Fields] OR "inhibitor sglt 2"[All Fields]) OR ("sodium glucose transporter 2 inhibitors"[Pharmacological Action] OR "sodium glucose transporter 2 inhibitors"[MeSH Terms] OR "sodium glucose transporter 2 inhibitors"[All Fields] OR "sglt 2 inhibitor"[All Fields])) AND ("heart failure"[MeSH Terms] OR ("heart"[All Fields] AND "failure"[All Fields]) OR "heart failure"[All Fields])) OR ("heart failure"[MeSH Terms] OR ("heart"[All Fields] AND "failure"[All Fields]) OR "heart failure"[All Fields] OR ("cardiac"[All Fields] AND "failure"[All Fields]) OR "cardiac failure"[All Fields]) OR ("heart failure"[MeSH Terms] OR ("heart"[All Fields] AND "failure"[All Fields]) OR "heart failure"[All Fields] OR ("myocardial"[All Fields] AND "failure"[All Fields]) OR "myocardial failure"[All Fields]) OR ("heart failure"[MeSH Terms] OR ("heart"[All Fields] AND "failure"[All Fields]) OR "heart failure"[All Fields] OR ("heart"[All Fields] AND "failure"[All Fields] AND "left"[All Fields] AND "sided"[All Fields]) OR "heart failure left sided"[All Fields]) OR ("heart failure"[MeSH Terms] OR ("heart"[All Fields] AND "failure"[All Fields]) OR "heart failure"[All Fields] OR ("heart"[All Fields] AND "failure"[All Fields] AND "left"[All Fields] AND "sided"[All Fields]) OR "heart failure left sided"[All Fields]) OR ("heart failure"[MeSH Terms] OR ("heart"[All Fields] AND "failure"[All Fields]) OR "heart failure"[All Fields] OR ("left"[All Fields] AND "sided"[All Fields] AND "heart"[All Fields] AND "failure"[All Fields]) OR "left sided heart failure"[All Fields]) OR ("heart failure"[MeSH Terms] OR ("heart"[All Fields] AND "failure"[All Fields]) OR "heart failure"[All Fields] OR ("left"[All Fields] AND "sided"[All Fields] AND "heart"[All Fields] AND "failure"[All Fields]) OR "left sided heart failure"[All Fields]) OR ("heart failure"[MeSH Terms] OR ("heart"[All Fields] AND "failure"[All Fields]) OR "heart failure"[All Fields] OR ("heart"[All Fields] AND "failure"[All Fields] AND "right"[All Fields] AND "sided"[All Fields]) OR "heart failure right sided"[All Fields]) OR ("heart failure"[MeSH Terms] OR ("heart"[All Fields] AND "failure"[All Fields]) OR "heart failure"[All Fields] OR ("heart"[All Fields] AND "failure"[All Fields] AND "right"[All Fields] AND "sided"[All Fields]) OR "heart failure right sided"[All Fields]) OR ("heart failure"[MeSH Terms] OR ("heart"[All Fields] AND "failure"[All Fields]) OR "heart failure"[All Fields] OR ("right"[All Fields] AND "sided"[All Fields] AND "heart"[All Fields] AND "failure"[All Fields]) OR "right sided heart failure"[All Fields]) OR ("heart failure"[MeSH Terms] OR ("heart"[All Fields] AND "failure"[All Fields]) OR "heart failure"[All Fields] OR ("right"[All Fields] AND "sided"[All Fields] AND "heart"[All Fields] AND "failure"[All Fields]) OR "right sided heart failure"[All Fields]) OR ("heart failure"[MeSH Terms] OR ("heart"[All Fields] AND "failure"[All Fields]) OR "heart failure"[All Fields] OR ("congestive"[All Fields] AND "heart"[All Fields] AND "failure"[All Fields]) OR "congestive heart failure"[All Fields]) OR ("heart failure"[MeSH Terms] OR ("heart"[All Fields] AND "failure"[All Fields]) OR "heart failure"[All Fields] OR ("heart"[All Fields] AND "failure"[All Fields] AND "congestive"[All Fields]) OR "heart failure congestive"[All Fields]) OR ("heart failure"[MeSH Terms] OR ("heart"[All Fields] AND "failure"[All Fields]) OR "heart failure"[All Fields] OR ("heart"[All Fields] AND "decompensation"[All Fields]) OR "heart decompensation"[All Fields]) OR ("heart failure"[MeSH Terms] OR ("heart"[All Fields] AND "failure"[All Fields]) OR "heart failure"[All Fields] OR ("decompensation"[All Fields] AND "heart"[All Fields]) OR "decompensation heart"[All Fields]) OR (("chronic"[All Fields] OR "chronical"[All Fields] OR "chronically"[All Fields] OR "chronicities"[All Fields] OR "chronicity"[All Fields] OR "chronicization"[All Fields] OR "chronics"[All Fields]) AND ("heart failure"[MeSH Terms] OR ("heart"[All Fields] AND "failure"[All Fields]) OR "heart failure"[All Fields])) OR (("symptomatic"[All Fields] OR "symptomatically"[All Fields] OR "symptomatics"[All Fields]) AND ("chronic"[All Fields] OR "chronical"[All Fields] OR "chronically"[All Fields] OR "chronicities"[All Fields] OR "chronicity"[All Fields] OR "chronicization"[All Fields] OR "chronics"[All Fields]) AND ("heart failure"[MeSH Terms] OR ("heart"[All Fields] AND "failure"[All Fields]) OR "heart failure"[All Fields])) OR ("heart failure, diastolic"[MeSH Terms] OR ("heart"[All Fields] AND "failure"[All Fields] AND "diastolic"[All Fields]) OR "diastolic heart failure"[All Fields] OR ("diastolic"[All Fields] AND "heart"[All Fields] AND "failure"[All Fields])) OR (("diastole"[MeSH Terms] OR "diastole"[All Fields] OR "diastoles"[All Fields] OR "diastolic"[All Fields] OR "diastolically"[All Fields]) AND ("dysfunctional"[All Fields] OR "dysfunctionals"[All Fields] OR "dysfunctioning"[All Fields] OR "dysfunctions"[All Fields] OR "physiopathology"[MeSH Subheading] OR "physiopathology"[All Fields] OR "dysfunction"[All Fields]))) AND (randomizedcontrolledtrial[Filter]) | 18,921 |
| EMBASE | **1** exp heart failure/  **2**  (Cardiac Failure or Myocardial Failure or Heart Failure, Left-Sided or Heart Failure, Left Sided or Left-Sided Heart Failure or Left Sided Heart Failure or Heart Failure, Right-Sided or Heart Failure, Right Sided or Right-Sided Heart Failure or Right Sided Heart Failure or Congestive Heart Failure or Heart Failure, Congestive or Heart Decompensation or Decompensation, Heart or Diastolic heart failure or Diastolic dysfunction or Chronic heart failure or symptomatic chronic heart failure).mp. [mp=title, abstract, heading word, drug trade name, original title, device manufacturer, drug manufacturer, device trade name, keyword heading word, floating subheading word, candidate term word]  **3**  exp sodium glucose cotransporter 2 inhibitor/  **4**  (Sodium-Glucose Transporter 2 Inhibitors or Sodium Glucose Transporter 2 Inhibitors or SGLT-2 Inhibitors or SGLT 2 Inhibitors or SGLT2 Inhibitors or Sodium-Glucose Transporter 2 Inhibitor or Sodium Glucose Transporter 2 Inhibitor or SGLT2 Inhibitor or Inhibitor, SGLT2 or Gliflozin or SGLT-2 Inhibitor or Inhibitor, SGLT-2 or SGLT 2 Inhibitor).mp. [mp=title, abstract, heading word, drug trade name, original title, device manufacturer, drug manufacturer, device trade name, keyword heading word, floating subheading word, candidate term word]  **5**  exp randomized controlled trial/  **6**  (control or Randomized).mp. [mp=title, abstract, heading word, drug trade name, original title, device manufacturer, drug manufacturer, device trade name, keyword heading word, floating subheading word, candidate term word]  **7**  1 or 2  **8**  3 or 4  **9**  5 or 6  **10**  7 and 8 and 9 | (658292)  (188726)  (28146)  (11632)  (807032)  (5890502)  (683059)  (29074)  (5891635)  (3402) |
| Cochrane | #1 MeSH descriptor: [Heart Failure] explode all trees  #2 (Cardiovascular NEXT (failure* or decompensation)):ti,ab,kw OR (Heart NEXT (failure* or decompensation)):ti,ab,kw OR (Symptomatic NEXT (heart failure* or cardiac failure*)):ti,ab,kw (Word variations have been searched)  #3 MeSH descriptor: [Sodium-Glucose Transporter 2 Inhibitors] explode all trees  #4 (#1 OR #2) AND #3 | 14671  36643  841  286 |
